# Severe Short Stature, Pathological Fracture, and Avascular Necrosis Due to Prolonged Over-the-Counter Steroid Use in an Adolescent Patient

**DOI:** 10.7759/cureus.99726

**Published:** 2025-12-20

**Authors:** Bushra Amer, Mohd Salman, Ahmad Alam

**Affiliations:** 1 Department of Internal Medicine, Berkshire Medical Center, Pittsfield, USA; 2 Department of Medicine, Jawaharlal Nehru Medical College, Aligarh Muslim University, Aligarh, IND; 3 Rajiv Gandhi Centre for Diabetes and Endocrinology, Jawaharlal Nehru Medical College, Aligarh Muslim University, Aligarh, IND

**Keywords:** avascular necrosis, cushing syndrome, delayed puberty, growth disorders, over-the-counter drugs, self-medication, severe osteoporosis

## Abstract

Glucocorticoids (GCs) are widely prescribed for their anti-inflammatory and immunosuppressive properties; however, prolonged, unsupervised, or over-the-counter (OTC) use, particularly during childhood and adolescence, can have profound consequences. We present a case of an 18-year-old male patient with a 10-year history of self-medicating with oral prednisolone for joint pain, without medical supervision. He presented with severe hip pain and difficulty ambulating. Examination revealed classical Cushingoid features, severe short stature, and delayed puberty. Pelvic radiographs revealed a pathological fracture of the left femoral neck and avascular necrosis (AVN) of the right femoral head. A dual-energy X-ray absorptiometry (DXA) scan demonstrated severe osteoporosis. Hormonal evaluation revealed suppressed 8:00 AM serum cortisol, consistent with secondary adrenal insufficiency. This case underscores the devastating consequences of unregulated GC use during adolescence, resulting in growth failure, delayed puberty, osteoporosis, adrenal insufficiency, and AVN. It highlights the need for clinician vigilance, public education, and policy-level regulation of OTC steroid access to prevent such avoidable endocrinopathies.

## Introduction

Glucocorticoids (GCs) are among the most widely used medications in clinical practice due to their potent anti-inflammatory and immunosuppressive properties [1]. Despite their therapeutic value, prolonged, unsupervised, or over-the-counter (OTC) use can lead to iatrogenic Cushing’s syndrome (CS), characterised by central adiposity, easy bruising, proximal muscle weakness, and metabolic disturbances [2]. In adults, iatrogenic CS often arises from extended or unsupervised steroid therapy, including OTC availability for conditions such as asthma, chronic obstructive pulmonary disease, rheumatologic disorders, and chronic dermatologic conditions like eczema or skin infections [3].

The consequences of chronic GC exposure are even more profound in children and adolescents, as this developmental period is critical for linear growth, pubertal maturation, and peak bone mass accrual. In this vulnerable age group, inappropriate steroid exposure may result in growth failure, delayed or arrested puberty, severe osteoporosis, and increased fracture risk [4]. We report a case of an adolescent patient with long-term unsupervised and OTC use.

GCs are used for joint pain in patients who present with severe short stature, pubertal arrest, profound osteoporosis with pathological fracture, and avascular necrosis (AVN) of the hip. This case illustrates the multisystem consequences of chronic steroid misuse in adolescents and emphasises the urgent need for clinician vigilance, public awareness, and stricter regulation of OTC GC sales.

## Case presentation

**Figure 1:**
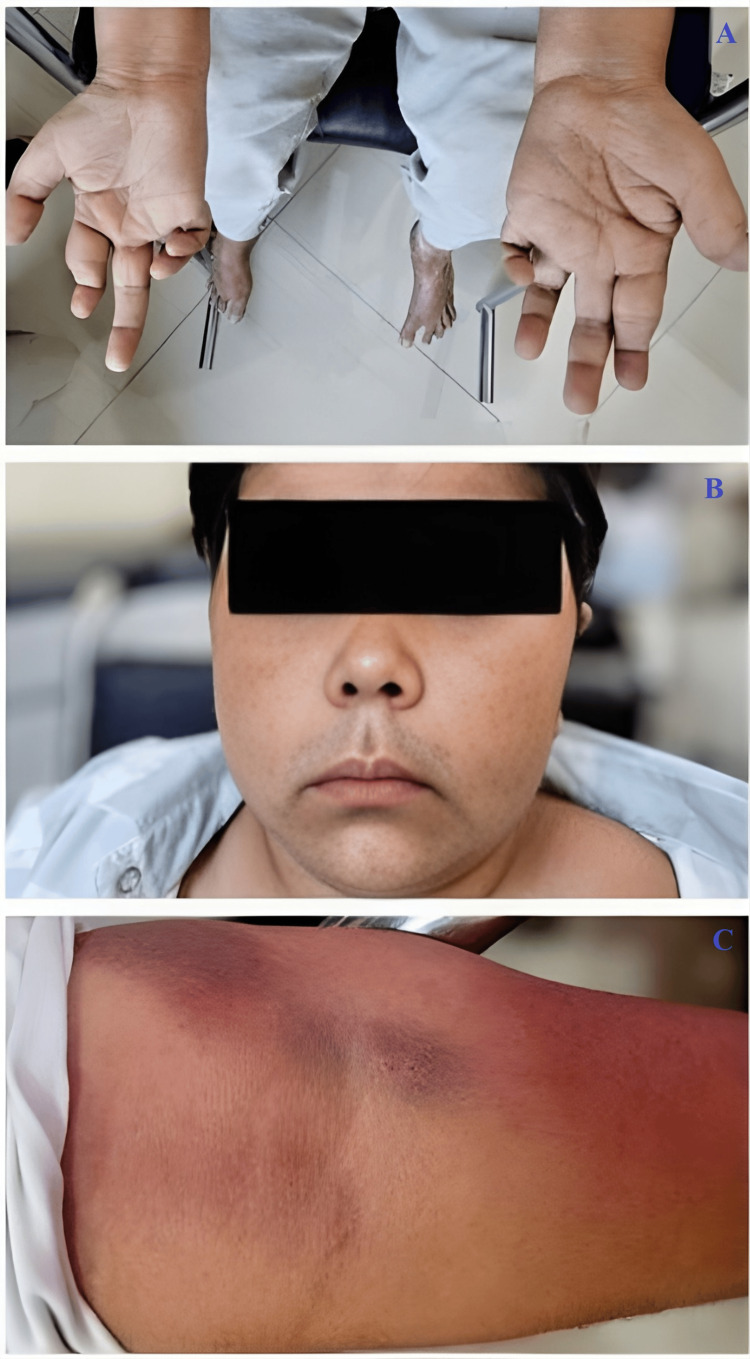
Clinical images showing finger deformities of both hands (A), moon facies consistent with chronic glucocorticoid exposure (B), and ecchymoses over the upper limb indicating easy bruising (C)

An 18-year-old adolescent presented with severe pain in both hips and difficulty walking. He had a decade-long history of self-medicating with OTC oral GCs for joint pain without medical supervision. On further inquiry, the patient admitted to taking oral prednisolone intermittently, often at doses of 10-20 mg/day, without tapering or medical follow-up. Although exact records were not available, the frequency and duration of intake suggest a cumulative GC exposure likely exceeding 30-50 grams of prednisolone equivalent over the past 10 years. On examination, he exhibited classic Cushingoid features such as moon facies, facial plethora, proximal muscle weakness, easy bruising, and dermal atrophy. He also had pain and tenderness in multiple small and large joints, along with finger deformities (Figure 1).

He had severe short stature (height: 120 cm; height SDS: -8.1), as per the Indian Academy of Paediatrics (IAP) growth charts and showed delayed puberty with sexual maturity rating A0P3 and bilateral testicular volume of 6 mL [5]. Laboratory evaluation revealed markedly elevated rheumatoid factor and anti-CCP antibody levels, supporting the diagnosis of juvenile idiopathic arthritis (JIA). Hormonal testing demonstrated decreased luteinizing hormone (LH), follicle-stimulating hormone (FSH), and total testosterone levels, consistent with hypogonadotropic hypogonadism. Additionally, his morning serum cortisol was significantly decreased, indicating adrenal suppression (Table 1).

**Table 1:** Biochemical characteristics of the patient CCP: cyclic citrullinated peptide, LH: luteinizing hormone, FSH: follicle-stimulating hormone, T3: triiodothyronine, T4: thyroxine, TSH: thyroid-stimulating hormone, ACTH: adrenocorticotropic hormone ^a^Low vitamin D with raised ALP suggests vitamin D deficiency-related secondary hyperparathyroidism. ^b^Low cortisol with inappropriately normal ACTH indicates secondary adrenal insufficiency

Parameter	Result	Reference Range
Haemoglobin (g/dL)	12.1	12-16
Erythrocyte sedimentation rate (mm/hr)	60	<20
C-Reactive Protein (mg/L)	70.6	<5
Anti-CCP (U/mL)	240	<17
Rheumatoid Factor (IU/mL)	180	<14
Serum calcium (mg/dL)	8.9	8.5-10.2
Serum phosphate (mg/dL)	2.7	2.5-4.5
Alkaline phosphatase (U/L)^a^	320	30-130
Vitamin D (25-OH, ng/mL)	12	30-100
LH (IU/L)	0.8	1.7-8.6
FSH (IU/L)	1.2	1.5-12.4
Testosterone (ng/mL)	0.8	3-8
T3 (ng/dL)	110	80 -200
T4 (µg/dL)	11.4	4.5- 12.5
TSH (µIU/mL)	2.2	0.3- 5.4
Serum cortisol, 8:00 (µg/dL)^b^	<1	5-22
Plasma ACTH (pg/mL)	12	10-65

**Figure 2:**
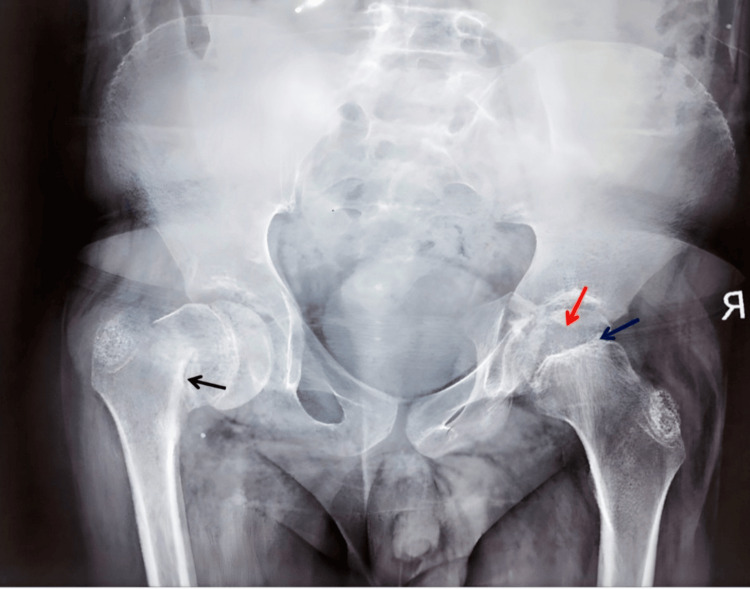
Pelvic radiograph showing a small, sclerosed femoral head (red arrow) with an irregular, sclerotic epiphysis (blue arrow), consistent with avascular necrosis, and a pathological fracture of the left femoral neck (black arrow)

A radiograph of the pelvis revealed a pathological fracture of the left femoral neck and AVN of the right femoral head (Figure 2).

Lumbar spine (L1-L4) bone mineral density (BMD), measured using dual-energy X-ray absorptiometry (DXA) on a Hologic system, showed a Z-score of -7.9, indicating bone density well below the expected range for age (Table 2).

**Table 2:** Bone mineral density report of the patient BMC: bone mineral content, BMD: bone mineral density

Region	Area (cm²)	BMC (g)	BMD (g/cm²)	Z-score
L1	7.59	2.61	0.343	-6.6
L2	7.96	3.10	0.390	-6.4
L3	7.84	3.40	0.434	-6.1
L4	8.78	2.89	0.329	-6.9
Total	32.17	12.00	0.373	-7.9

The clinical picture was consistent with iatrogenic CS due to chronic GC excess, leading to secondary adrenal insufficiency, profound bone demineralisation, and delayed pubertal development. The patient was counselled to discontinue further unsupervised GC use immediately. In view of markedly suppressed morning serum cortisol (<1 µg/dL), he was initiated on physiological GC replacement with oral hydrocortisone (10 mg/m²/day in divided doses) and educated regarding stress dosing, sick day management, and the need for gradual hypothalamic-pituitary-adrenal axis monitoring. For low BMD for age (Z-score -7.9) and a history of pathological fracture, teriparatide (20 µg subcutaneous daily) was initiated. He was also supplemented with elemental calcium 1,000 mg/day and vitamin D₃ 600,000 IU weekly for 12 weeks, followed by a maintenance dose thereafter. Orthopaedic management was planned, including internal fixation of the left femoral neck fracture using cannulated cancellous screws and core decompression of the right femoral head for AVN. Given delayed puberty, pubertal progression was to be monitored, with testosterone replacement considered if spontaneous development did not occur. He was referred to rheumatology for initiation of steroid-sparing immunomodulatory therapy for JIA.

## Discussion

**Figure 3:**
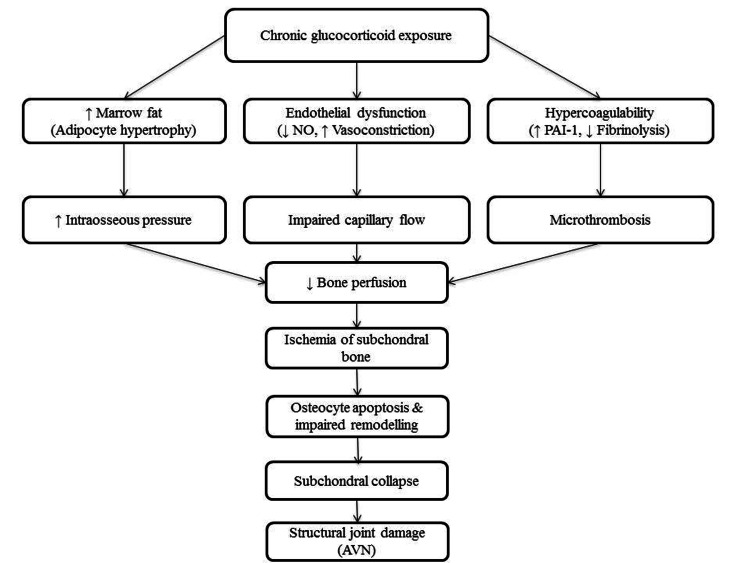
Flowchart illustrating the mechanism of glucocorticoid-induced avascular necrosis Image Credits: Bushra et al. Figure created by authors using Microsoft PowerPoint (Microsoft Corporation, Redmond, WA)

This case underscores the multisystem impact of prolonged, unsupervised GC exposure during critical developmental windows in adolescence. The endocrine sequelae observed in our patient, growth failure, delayed puberty, severe osteoporosis, and secondary adrenal insufficiency, highlight the need for increased awareness and regulatory vigilance in contexts where OTC steroid use remains prevalent. GCs profoundly impact linear growth and pubertal development, particularly when used chronically during childhood and adolescence. In our patient, who had a height of 120 cm (height SDS: -8.1), prolonged unsupervised GC use led to severe growth retardation. GCs inhibit growth hormone (GH) secretion at both hypothalamic and pituitary levels, reduce hepatic IGF-1 synthesis, and impair IGF-1 signalling at the growth plate. Additionally, they increase chondrocyte apoptosis, suppress collagen synthesis, and directly interfere with epiphyseal plate integrity, resulting in reduced growth velocity and compromised final height [6,7]. GCs also suppress the hypothalamic-pituitary-gonadal (HPG) axis by inhibiting GnRH pulsatility and reducing LH and FSH secretion [8]. Our patient had delayed puberty, with a sexual maturity rating of A0P3 and bilateral testicular volume of 6 mL, suggestive of early pubertal onset followed by arrest. In addition to GC exposure, the underlying JIA also contributed to growth failure and delayed puberty through elevated proinflammatory cytokines, resulting in functional suppression of the hypothalamic-pituitary-growth and HPG axes. One of the most serious consequences of chronic GC exposure is GC-induced osteoporosis, which results from increased osteoclastic activity, reduced osteoblast number and function, impaired calcium absorption, and secondary hyperparathyroidism [9]. Adolescents are particularly vulnerable, as peak bone mass accrual is compromised. In our patient, DXA revealed a Z-score of -7.9 at the lumbar spine, far below age- and sex-matched norms, placing him at extreme fracture risk. The presence of a pathological femoral neck fracture and contralateral AVN further underscores the skeletal morbidity. AVN of the femoral head is a well-recognised complication of prolonged GC exposure and can be further potentiated by chronic inflammatory conditions such as JIA. GCs impair bone perfusion through several mechanisms: they promote lipid deposition within arterioles, increase intraosseous pressure through marrow fat hypertrophy, and induce endothelial dysfunction, all of which reduce perfusion to the femoral head, a site particularly vulnerable due to its terminal blood supply [10]. Steroids also upregulate procoagulant factors and reduce fibrinolytic activity, further predisposing to microvascular thrombosis. These changes can cumulatively disrupt the subchondral circulation, ultimately resulting in ischemic necrosis of bone and collapse of the articular surface (Figure 3).

Clinically, AVN often presents insidiously with groin or thigh pain and progressive loss of joint function. Radiographically, early disease may only be detected on MRI, but in advanced cases, such as in our patient, plain radiographs reveal femoral head flattening, sclerosis, and joint space narrowing. Once structural collapse occurs, the prognosis is poor, and conservative measures are largely ineffective [10]. In India and similar settings, oral corticosteroids are easily obtained and often misused for minor ailments or general well-being [11]. Low public awareness and weak regulatory oversight allow this misuse to continue, especially in children. The resulting endocrine harm underscores the need for stricter control of steroid sales, stronger community education, and better training to recognise early iatrogenic CS [12].

## Conclusions

This case exemplifies the grave consequences of unregulated access to potent GCs in low- and middle-income countries. In many such settings, including India, oral corticosteroids are readily available over the counter without prescription, often misused for musculoskeletal complaints or perceived general well-being. Public awareness of the long-term adverse effects of steroid use, especially in children and adolescents, is limited. There is also a lack of regulatory enforcement, pharmacist accountability, and physician-driven education at the primary care level. The cumulative impact can lead to long-term endocrine sequelae, as seen in our patient, including growth failure, delayed puberty, adrenal suppression, and osteoporosis with pathological fractures. From a public health standpoint, there is an urgent need for policy-level interventions to restrict unsupervised steroid dispensing, community education campaigns to raise awareness about steroid toxicity, and training of frontline healthcare providers to recognise early signs of iatrogenic CS. Strengthening pharmacovigilance and involving both public and private sector stakeholders can help curb such preventable endocrinopathies.
